# The effectiveness of a mobile application for the development of palpation and ultrasound imaging skills to supplement the traditional learning of physiotherapy students

**DOI:** 10.1186/s12909-016-0775-1

**Published:** 2016-10-19

**Authors:** Carolina Fernández-Lao, Irene Cantarero-Villanueva, Noelia Galiano-Castillo, Elena Caro-Morán, Lourdes Díaz-Rodríguez, Manuel Arroyo-Morales

**Affiliations:** 1 Physical Therapy Department, Instituto Biosanitario Granada (IBS.Granada), Instituto Mixto Deporte y Salud (iMUDS), University of Granada, Granada, Spain; 2 Nursing Department, Instituto Biosanitario Granada (IBS.Granada), University of Granada, Granada, Spain; 3 Physical Therapy Department, Faculty of Health Sciences, University of Granada, Avda. de la Ilustración 60, 18071 Granada, Spain

**Keywords:** Teaching/learning strategies, M-learning, Physical therapy

## Abstract

**Background:**

Mobile learning (m-learning) has becoming very popular in education due to the rapidly advancing technology in our society. The potentials of the mobile applications should be used to enhance the education process. Few mobile applications have been designed to complement the study of physical therapy skills for physiotherapy students. The aim of this study was to investigate whether a mobile application, as a supplement to traditional learning, is useful for physiotherapy students in the acquisition of palpation and ultrasound skills in the shoulder area.

**Methods:**

Forty-nine students participated in this single-blinded, randomized controlled study. They were randomly distributed into two groups: experimental, with free access to the mobile application; and control, with access to traditional learning materials on the topic. Objective structured clinical evaluation (OSCE) and multiple-choice questionnaire (MCQ) were used to assess the educational intervention. Then, we also assessed the time taken to get a reliable ultrasound image and to localize a specific shoulder structure by palpation.

**Results:**

There was no significant intergroup difference in the acquisition of theoretical knowledge (*p* = .089). Scores were significantly higher in the experimental group than in the control group for the majority items in the ultrasound assessment; positioning of patient (*p* < .001), positioning of ultrasound probe (*p* = 0.007), handling of ultrasound probe (*p* = .013) and global OSCE (*p* < .001) and skills in palpation of the shoulder; position of patient (*p* = .009), direction of palpation contact (*p* = .021) and global OSCE (*p* = .034). There were no significant differences in the time required to perform the examination between groups in ultrasound (*p* = .944) and palpation (*p* = .393). The results from the post-program survey assessing the global satisfaction with the mobile application were high (8.200 ± .767), on an 11 numeric point rating scale.

**Conclusion:**

These results suggest the effectiveness of an m-learning program as a complement to traditional education for developing skills in ultrasound and palpation of the shoulder region in undergraduate physiotherapy students.

**Electronic supplementary material:**

The online version of this article (doi:10.1186/s12909-016-0775-1) contains supplementary material, which is available to authorized users.

## Background

Rapidly advancing technology has led to the rebirth of personal computers in the form of smartphones and tablets [1]. These devices have become very popular in our society in a very short time, and their use in the classroom is increasingly [2]. Mobile learning, also known as “m-learning”, providesmodern methodsto support theteaching-learningprocessby usingdifferentmobile devices, such as laptops, iPods, tablets and smartphones [3]. M-learning is defined as “theability to accesseducational resources, tools and materialsanywhereand everywhere, usinga mobile device” [4]. This type of learning is portable, personalized, collaborative, and interactive, and it presents different characteristics thantraditional learning because instructioncan be doneanywhere, at any time and with an emphasis on the importance of accessto knowledgeat the right time [3]. Today, learners are continually on the move, taking ideas and learning resources from a specific location and developing them in another location [5]. This potential should beused to combineacademic lifeand the socio-economicdemands of the moment, specifically in the presence of current economic difficulties. This type of learning has received considerable attention in the educational environment. Wu, et al. (2012) [6] found that the majority of the studies that assessed m-learning presented positive outcomes for the learning process. In a health education context, and specifically in physiotherapy, the use of mobile devices could be useful for reinforcing different skills related to the diagnostic competence developed in the profession.

Currently, numerous applications are available forprofessionalimagingdiagnosis onthe differentmobile platforms. Following Székely et al. (2013) [1], these applicationscan be categorized intodecision support, diagnosis, medical books, interactive encyclopaedias, document organization software andjournal readers. These applications could provide new perspectives for practitioners in diagnostic imaging or could be used for reference, learning, consultation and communication with patients [1]. A previous study [7] examined the efficacy of a 3D mobile phone application to teach manual therapy skills, and the authors found it useful for physiotherapy students. However, to our knowledge, few mobile applications have been designed to complement the study of physical therapy skills with a specific design for physiotherapy students.

Many physiotherapists are adding musculoskeletal ultrasound to their daily practice and are among the many healthcare professionals studying the potential clinical integration of this technology [8]. Furthermore, ultrasound imaging has been proven to be a useful toolfor the diagnosisof painand functional impairmentof the shoulder joint [9–11], and it isbecoming awidely used toolfor assessing this anatomical region. Advances intechnology anda better understanding ofthe pathologyand anatomymakeultrasoundone of the mostuseful tests, especially in the handsof experienced professionals. Although the application of ultrasound imaging in E-learning has been explored in previous studies [12–14], we aimed to develop this type of experience specifically in an m-learning environment.

Thus, the aim of this study was to investigate whether a mobile application, as a supplement to traditional learning, is useful for physiotherapy students in the acquisition of palpation and ultrasound skills in the shoulder area.

## Methods

A single-blinded, randomized controlled study was carried out in volunteer students from the degree of Physiotherapy in Health Sciences Faculty of the University of Granada. The proposed methodology was conducted during the first semester of the 2013–14 academic year and introduced the use of a mobile application in the study of musculoskeletal assessment competencies. Participants were recruited through public announcement at the university. All of the volunteers were enrolled in the study unless they reported previous knowledge/training in musculoskeletal ultrasound imaging in a pre-enrolment questionnaire. All participants had similar levels of knowledge concerning anatomy, physiology and biomechanics. During the first talk, the volunteers were clearly informed that the assessments of their performance in the current study would have no effects on their course evaluation or grades. The ethics approval for study was granted by the Educational Innovation Unit Committee of the University of Granada, Spain (PID 13–86) and was conducted in accordance with the Helsinki Declaration. All students signed the informed consent that was required to participate in the study.

**Fig. 1:**
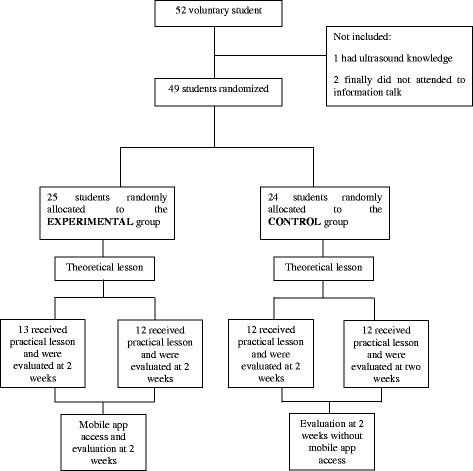
Participants flow-chart

The procedure was conducted in the Physical Therapy Laboratory of the Faculty by 3 professors and 3 teaching fellows; a teacher:student ratio of 1:6–8 was thus obtained. All participants received theory and practice traditional training in ultrasound imaging and palpation of the musculoskeletal region of the shoulder in the same module of the study. The educational program had 6 contact learning lessons and 20 self-study hours that were focused on the theoretical and practical learning about palpation and ultrasound imaging procedures in the shoulder region. The students were randomly divided in two groups for the 2-h theoretical lessons using a computer-generated number sequence. Teachers were blinded to the participant allocation group. Then these two groups were divided into two parts (2 groups in the experimental and 2 in the control group) for the 4-h practical lessons to facilitate the learning process. A participant flow-chart describes the procedure (Fig. 1).

The outcome measures were assessed in the two groups of study (m-learning and control group). The palpation skills sessions were conducted by the professors according to the method previously described by Tixa (2006) [15], and the ultrasound imaging sessions were developed according to a previously reported methodology [16]. The 20 self-study hours were carried out by the students on their own using the mobile app or traditional study models (e.g., e-books, books, or journal papers available in the University Library), depending on the study group. The ultrasound device used by the participants was the same model for all participants: a 12-MHz linear probe (MyLab 25; Esaote Medical Systems, Genova, Italy).

Both groups had two weeks to study after the on-campus session, but the m-learning group received this session after the control group completed the evaluation to avoid encouraging this group to seek information using the Internet or accessing to the mobile app.

**Fig. 2:**
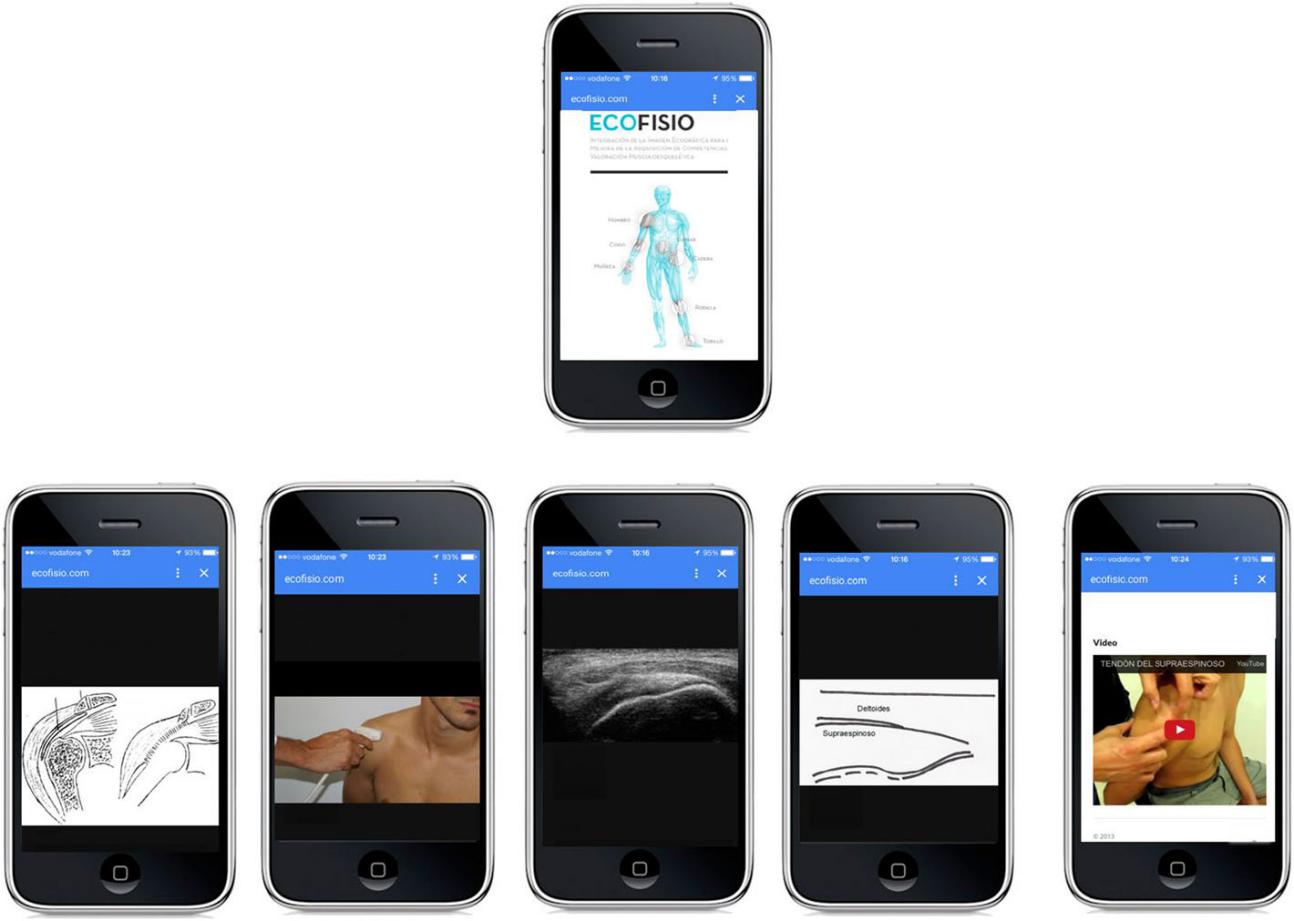
View of the mobile application screens from the main menu to the drawing of the anatomical description, the placement of the ultrasound probe, the ultrasound slice, the diagram of the ultrasound image and the video of the manual palpation procedure

The mobile application has specific content based on the specific region of the shoulder being studied. For each structure of the shoulder, there exists a theoretical description, a drawing with the anatomical description, an image with the specific placement of the ultrasound probe, an ultrasound slice, a diagram of the ultrasound image and a video of the manual palpation procedure (Fig. 2).

### Outcome measures

The theoretical knowledge was evaluated using a multiple choice questionnaire (MCQ) that contained 20 questions and had a maximum of 10 points. The skills were assessed using an objective structured clinical evaluation (OSCE) with two components. The ultrasound component consisted of positioning the patient, positioning the ultrasound probe, the orientation of the ultrasound probe, handling the ultrasound probe, and image adjustment. The palpation component consisted of positioning the patient, positioning the extremity, the direction of palpation contact and the precision of palpation. To assess each of these items, we used a qualitative grading system that ranged from 3 = excellent to 0 = incorrect. At the end, maximum scores (15 and 12 points each) for ultrasound and palpation skills were obtained. The assessment was developed by two physiotherapists who were experienced in ultrasound and palpation and were blinded to the subjects’ groups. Each participant performed the exam on the same human model.

In addition, the participants assessed the quality of the intervention using a 5-point Likert scale (5 = strongly agree, 1 = strongly disagree) [14, 17]. The m-learning group assessed the mobile application by rating their global satisfaction using an 11 numeric point rating scale (ranging from 10 = totally satisfied to 0 = totally unsatisfied).

### Sample size calculation

The estimated sample size was 20 participants in each group, based on a previous study [14], which would provide a power of 90 % to detect a significant mean difference of 3.5 (3) points in the palpation assessment (OSCE), assuming a type 1 error (α) of 5 % and a type 2 error (β) of 10 %. Considering a drop-out rate of 20–30 %, we decided to enrol 24–25 subjects per group. Before the on-campus sessions, the participants were assigned randomly to each group by an independent researcher using the EPIDAT 3.1 software (Xeral de SaúdePública, La Coruña, Spain).

### Statistical analysis

Data were analyzed using IBM SPSS Statistics, version 22.0. The results are expressed as the mean, standard deviation (SD) and confidence interval (95 % CI). Because the Kolmogorov-Smirnov test revealed a normal distribution (*P* > .05) of OSCE and MCQ, we applied Student’s *t* test. The non-parametric Mann–Whitney U test was used to analyze the differences in the OSCE time. The statistical analysis was conducted at a 95 % confidence level, and a *P* value less than .05 was considered statistically significant.

## Results

Forty-nine students participated in the study. The sample was composed of twenty-six (53.1 %) women and twenty-three (46.9 %) men. Twenty-five students (20.720 ± 6.148 years) were allocated in the experimental group, and twenty-four (18.880 ± 1.849 years) were in the control group, and these groups did not differ in age (*p* = .165), gender (*p* = .056) or previous knowledge of shoulder anatomy (*p* = .699).

Within the ultrasound skills, there were statistically significant differences between the groups in the positioning of patient (*p* < .001), where the experimental group showed better results compared with the control group. The group with the mobile app also positioned the ultrasound probe better than the control group did (*p* = .007), and they demonstrated better handling of ultrasound probe (*p* = .013). The differences between the groups in the orientation of the ultrasound probe (*p* = .548) and the image adjustment (*p* = .191) were not statistically significant.

With respect to palpation skills, the experimental group showed better positioning of the patient (*p* = .009) and a better direction of palpation (*p* = .021). They did not have significant differences in the position of the extremity (*p* = .521) or the precision of palpation (*p* = .116). The global scores for the OSCE components were significantly higher in the experimental group than in the control group for both ultrasound (*p* < .001) and palpation skills (*p* = .034). There were no significant differences in the time required to perform the examination between the groups. Results are detailed in Table 1.

**Table 1:** Comparison of test results between the M-learning and control groups

Variable	M-learning group (*N* = 25)	Control group (*N* = 24)	*P* value
Knowledge test (maximum 10 points)	7.21 ± 1.988	8.09 ± .921	.089
OSCE- time ultrasound (sec)	56.756 ± 27.563	51.985 ± 28.726	.944
OSCE-time palpation (sec)	41.223 ± 17.757	45.926 ± 20.817	.393
Ultrasound skills			
- Positioning of patient	2.925 ± .266	1.869 ± .344	.000*
- Positioning of ultrasound probe	2.629 ± .791	1.869 ± 1.099	.007*
- Orientation (angle) of ultrasound probe	2.407 ± .930	2.227 ± 1.151	.548
- Handling of ultrasound probe	1.703 ± .724	1.173 ± .716	.013*
- Image adjustment	2.333 ± 1.000	1.913 ± 1.239	.191
- Global OSCE (maximum 15 points)	12.000 ± 2.572	9.000 ± 2.943	.000*
Palpation skills			
- Position of patient	3.000 ± .000	2.708 ± .55	.009*
- Position of extremity	2.461 ± .904	2.291 ± .954	.521
- Direction of palpation contact	2.461 ± .859	1.833 ± 1.007	.021*
- Precision of palpation	1.846 ± .880	1.416 ± 1.017	.116
- Global OSCE (maximum 12 points)	12.038 ± 3.155	9.833 ± 3.963	.034*

*Significant differences between groups (Student’s t test and Mann-Witney U test).

Values ± SD are expressed as the mean (95 % confidence interval)

**Fig. 3:**
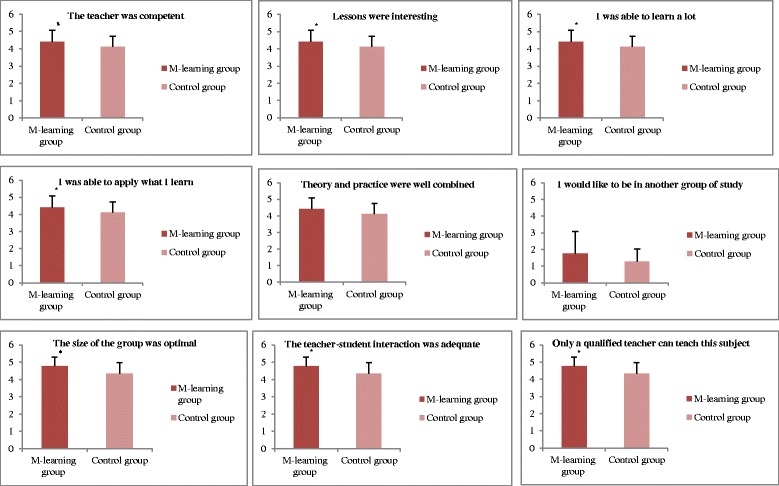
Comparison between groups responses on the post-program participant survey

Figure 3 shows the results from the between-group comparison for the assessment of the learning process during the study. There were higher ratings in the responses from the m-learning group compared with the control group for the items the teacher was competent (*p* < .001), lessons were interesting (*p* = .016), I was able to learn a lot (*p* = .022), the size of the groups was optimal (*p* = .002) and the teacher-student interaction was adequate (*p* = .008). There were no significant differences between groups for the items theory and practice were well combined (*p* = .114) and I would like to have been in another learning group (*p* = .185).

Finally, the results from the post-program survey assessing the global satisfaction from the m-learning group with the mobile application were high (8.200 ± .767), on an 11 numeric point rating scale.

## Discussion

The results of the study suggest that a mobile application can be useful for reinforcing the traditional learning that is used to acquire palpation and ultrasound skills in physiotherapy students. We used an m-learning process to complement the study of the shoulder region based on palpation and ultrasound skills. These findings are in line with prior works on the positive use of new technologies in the study of manual therapy [7, 14, 18, 19].

The students in the m-learning group had better results for nearly all of the components of the ultrasound assessment, which is in accordance with previous work that was carried out using a similar methodology but was conducted in an e-learning environment [17]. This trend, however, was not followed for the image adjustment and orientation of the ultrasound probe. This difference may have occurred because the ability to acquire a good ultrasound image can be one of the most difficult parts of the ultrasound evaluation, and it should be practiced more frequently in an on-campus setting. This fact is in line with Knobe et al. [20] teaching of some manual procedures by an experienced instructor is a basic prerequisite for goal-orientated training. The results for the palpation skills in general were also better in the m-learning group, except for the position of the extremity and the precision of palpation, which is in agreement with a previous study [14]. However, this finding was not similar to that of Cantarero-Villanueva (2012) [17], who had better scores for these items in the e-learning group. The students may thus need more face-to-face preparation to develop good precision for palpation, or there may be a need to improve the m-learning strategies for these specific issues, considering the difficulty of palpation on the shoulder area because of the great number of bony landmarks, joints and soft tissues involved.

We did not find significant differences in the time required to perform the evaluations, but it was better in the m-learning group for the palpation and was surprisingly better in the control group for the ultrasound exam. It is possible that the students in the m-learning group spent more time positioning the probe to acquire the ultrasound image of the specific structure because they were more motivated to acquire a good result in the ultrasound assessment. The required time to develop the assessments was not a primary outcome of the current study, considering our main objective to improve the skills in ultrasound imaging and palpation.

Furthermore, the responses to the post-program survey were better in the m-learning group than in the control group, as expected. This result agrees with previous works that used an e-learning strategy for improving palpation and ultrasound skills [14, 17]. In general, the assessment was very positive, which is similar to another study that used an m-learning proposal to improve manual therapy skills [7]. These outcomes could highlight the current familiarization of the students with the new technologies [21], support the use of mobile devices to complement the study at the university level, and bring attention to self-learning [7] and the integration of mobile technologies as a way to facilitate constructivist learning [22].

Some limitations should be considered in the present study. First, the experience was carried out in a single faculty in one university, so the results could not be extrapolated to other populations, in different grades or with different languages. New experiences need to be carried out in different student populations (e.g., students from other faculties, other knowledge areas or countries). It would be interesting to develop video recordings for the ultrasound assessment, in a similar manner as for palpation, to facilitate the learning process of this part of the study. Finally, because the experience was carried out in an education course, it is possible that the students were more motivated to explore new learning strategies. It would be interesting to develop similar experiences in a different setting, where the motivations and backgrounds of students were entirely independent. Despite these limitations, the present work lays the groundwork for future studies in similar areas of health sciences to develop mobile applications that are useful in supporting the learning process.

## Conclusion

The results of the current study suggest the effectiveness of an m-learning program as a complement to on-campus education for developing skills in ultrasound and palpation of the shoulder region in undergraduate physiotherapy students, despite no significant differences were observed in theoretical knowledge but there existed impact of m-learning on the achievement of physical examination skills.

## Additional files

Additional file 2: Assessment questionnaire of the learning process. (DOCX 15 kb)
Additional file 3: Database. (XLSX 14.6 kb)
