# Supplementary material for: The effectiveness of a mobile application for the development of palpation and ultrasound imaging skills to supplement the traditional learning of physiotherapy students
Source: BMC Med Educ. 2016 Oct 19;16:274. doi: 10.1186/s12909-016-0775-1 (PMC5070141; doi:10.1186/s12909-016-0775-1)
Supplement: Additional file 2: — Assessment questionnaire of the learning process. (DOCX 15 kb) [file 12909_2016_775_MOESM2_ESM.docx]

**ASSESSMENT QUESTIONNAIRE OF THE LEARNING PROCESS**

**Please circle the number that corresponds better to your answer to each item.**

(1=strongly disagree, 5=strongly agree)

1. **The teacher was competent**

1 2 3 4 5

1. **Lessons were interesting**

1 2 3 4 5

1. **I was able to learn a lot**

1 2 3 4 5

1. **I was able to apply what I learn**

1 2 3 4 5

1. **Theory and practice were well combined**

1 2 3 4 5

1. **I would like to be in another group of study**

1 2 3 4 5

1. **The size of the group was optimal**

1 2 3 4 5

1. **The teacher-student interaction was adequate**

1 2 3 4 5

1. **Only a qualified teacher can teach this subject**

1 2 3 4 5
